# GPS-pPLM: A Language Model for Prediction of Prokaryotic Phosphorylation Sites

**DOI:** 10.3390/cells13221854

**Published:** 2024-11-08

**Authors:** Chi Zhang, Dachao Tang, Cheng Han, Yujie Gou, Miaomiao Chen, Xinhe Huang, Dan Liu, Miaoying Zhao, Leming Xiao, Qiang Xiao, Di Peng, Yu Xue

**Affiliations:** 1 Department of Bioinformatics and Systems Biology, MOE Key Laboratory of Molecular Biophysics, Hubei Bioinformatics and Molecular Imaging Key Laboratory, College of Life Science and Technology, Huazhong University of Science and Technology, Wuhan 430074, China; zchi@hust.edu.cn (C.Z.); tangdc@hust.edu.cn (D.T.); hancheng@hust.edu.cn (C.H.); d202180726@hust.edu.cn (Y.G.); d202180735@hust.edu.cn (M.C.); huangxinhe@hust.edu.cn (X.H.); begoniar@hust.edu.cn (D.L.); m202372738@hust.edu.cn (M.Z.); lemingxiao021106@163.com (L.X.); 2 School of Artificial Intelligence and Automation, Huazhong University of Science and Technology, Wuhan 430074, China; xq1620128@126.com

**Keywords:** posttranslational modification, phosphorylation, language model, deep learning, prokaryote

## Abstract

In the prokaryotic kingdom, protein phosphorylation serves as one of the most important posttranslational modifications (PTMs) and is involved in orchestrating a broad spectrum of biological processes. Here, we report an updated online server named the group-based prediction system for prokaryotic phosphorylation language model (GPS-pPLM), used for predicting phosphorylation sites (p-sites) in prokaryotes. For model training, two deep learning methods, a transformer and a deep neural network, were employed, and a total of 10 sequence features and contextual features were integrated. Using 44,839 nonredundant p-sites in 16,041 proteins from 95 prokaryotes, two general models for the prediction of *O*-phosphorylation and *N*-phosphorylation were first pretrained and then fine-tuned to construct 6 predictors specific for each phosphorylatable residue type as well as 134 species-specific predictors. Compared with other existing tools, the GPS-pPLM exhibits higher accuracy in predicting prokaryotic *O*-phosphorylation p-sites. Protein sequences in FASTA format or UniProt accession numbers can be submitted by users, and the predicted results are displayed in tabular form. In addition, we annotate the predicted p-sites with knowledge from 22 public resources, including experimental evidence, 3D structures, and disorder tendencies. The online service of the GPS-pPLM is freely accessible for academic research.

## 1. Introduction

Protein phosphorylation is an important regulatory mechanism highly conserved from prokaryotes to eukaryotes that orchestrates a broad spectrum of intracellular signaling pathways [1,2,3,4]. Mechanistically, the phosphate group of adenosine triphosphate (ATP) catalyzed by protein kinases (PKs) is covalently conjugated to specific amino acid residues of protein substrates, which results in the shaping of diverse aspects of targeted protein function, including activity, localization, and stability [5,6,7]. Phosphorylation was first discovered and widely investigated in eukaryotes, and increasing attention has been given to prokaryotic phosphorylation due to the rapid advancement of large-scale detection technology for protein posttranslational modifications (PTMs) [5,8,9]. In prokaryotic organisms, the majority of identified phosphorylation events are *O*-phosphorylation modifications, which mainly occur on serine (S), threonine (T), and tyrosine (Y) residues [10]. In addition, *N*-phosphorylation has also been detected on histidine (H), arginine (R), and lysine (K) residues [11,12]. Previous studies have revealed that several conserved motifs located in phosphorylated substrates are recognized and modified by eukaryotic-like protein kinases (ELKs) in prokaryotes, supporting the evolutionary conservation of phosphorylation [5,8,9]. In particular, prokaryotic phosphorylation is closely linked to many physiological processes, such as the cell cycle, cellular metabolism, and antibiotic persistence and virulence [5,13,14]. Thus, the identification of phosphorylation sites (p-sites) is fundamental for understanding their biological function and the regulatory mechanism of prokaryotic phosphorylation [15]. It also provides potential antibacterial targets for the treatment of infectious diseases [14,16].

The biochemical experimental methods for the identification of p-sites are time-consuming, laborious, and low-throughput. In recent years, mass spectrometry (MS)-based proteomic techniques have been adopted for the large-scale identification of prokaryotic p-sites [6,17,18]. For example, Potel et al. systematically identified 2129 p-sites from phosphoproteomic profiling of *Escherichia coli* [18]. Besides experimental approaches, eight computational tools have been developed for predicting *O*-phosphorylation or *N*-phosphorylation in prokaryotes [19,20,21] (Supplementary Table S1). NetPhosBac, the first bacteria-specific protein *O*-phosphorylation predictor, used the artificial neural network method, with a training dataset of 103 phosphoserine (pS) sites and 37 phosphothreonine (pT) sites, after homology reduction [20]. In 2019, MPSite took 1704 pS sites and 1401 pT sites from dbPSP [22] and used a random forest (RF) classifier to predict prokaryotic p-sites based on seven types of sequence features [19]. Later, Wang et al. compiled a large benchmark dataset from dbPSP 2.0 [23], containing 6629 pS sites, 5029 pT sites, and 3167 phosphotyrosine (pY) sites. They combined a capsule network and a self-attention mechanism to develop a tool for the prediction of prokaryotic *O*-phosphorylation [21]. Other tools included cPhosBac [24], prkC-PSP [25], RotPhoPred [26], PROSPECT [27], and pHisPred [28], and the latter two were implemented for predicting histidine *N*-phosphorylation in prokaryotes. Currently, the online services of only NetPhosBac and MPSite are active and can be freely accessed.

More recently, the transformer architecture with a self-attention mechanism has been utilized to extract the contextual features of protein sequences [29,30,31]. For example, LMPhosSite was developed by integrating per-residue contextualized embedding from a pretrained protein language model (PLM), ProtT5 [32], for the general prediction of eukaryotic p-sites [33]. Meanwhile, PhosphoLingo incorporated the contextual features generated from various PLMs, such as Evolutionary-Scale Modeling (ESM) [34] and ProtT5 [32], to predict eukaryotic p-sites [35]. In addition, several other models have been constructed by utilizing the contextual information extracted from protein sequences for p-site prediction in eukaryotes, including DL-SPhos [36], PTransIPs [37], Phosformer [38], PhosBoost [39], and PhosBERT [40]. However, it is still unclear whether integrating contextual features from language models would be helpful for the prediction of p-sites in prokaryotic organisms.

In this study, we first provide a brief review of the currently available prokaryotic phosphorylation prediction tools. We subsequently developed an online service named the group-based prediction system for prokaryotic phosphorylation language model (GPS-pPLM) to computationally analyze *O*- or *N*-phosphorylation sites in prokaryotes. We carefully collected and re-curated 44,839 nonredundant p-sites in 16,041 proteins across 95 species. Using the timestamp method, 90% of the p-site datasets were taken as the training data, and the remaining data served as independent testing data. In the process of model training, we employed two machine learning approaches, namely, the transformer and deep neural network (DNN), and combined eight sequence-based features as well as two contextual features from language models. To enhance the performance of the predictors, two general models were pretrained for the prediction of *O*-phosphorylation and *N*-phosphorylation, respectively, and then fine-tuned to construct 6 residue-specific predictors and 134 species-specific predictors. Compared with other available tools, including NetPhosBac [20] and MPSite [19], the GPS-pPLM shows a higher accuracy for the prediction of prokaryotic *O*-phosphorylation p-sites. The web service of GPS-pPLM allows users to submit one or multiple protein sequences in FASTA format or UniProt accession numbers, and the predicted results are displayed in tabular form. In addition, we also utilize information from 22 public resources to annotate the analyzed p-sites, including experimental evidence, 3D structures, and disorder tendencies. Overall, we anticipate that the GPS-pPLM could be a useful tool for identifying functional p-sites in prokaryotes. The online service of the GPS-pPLM is freely accessible for academic research at: https://pplm.biocuckoo.cn (accessed on 6 November 2024).

## 2. Materials and Methods

### 2.1. Data Collection and Preparation

**Figure 1:**
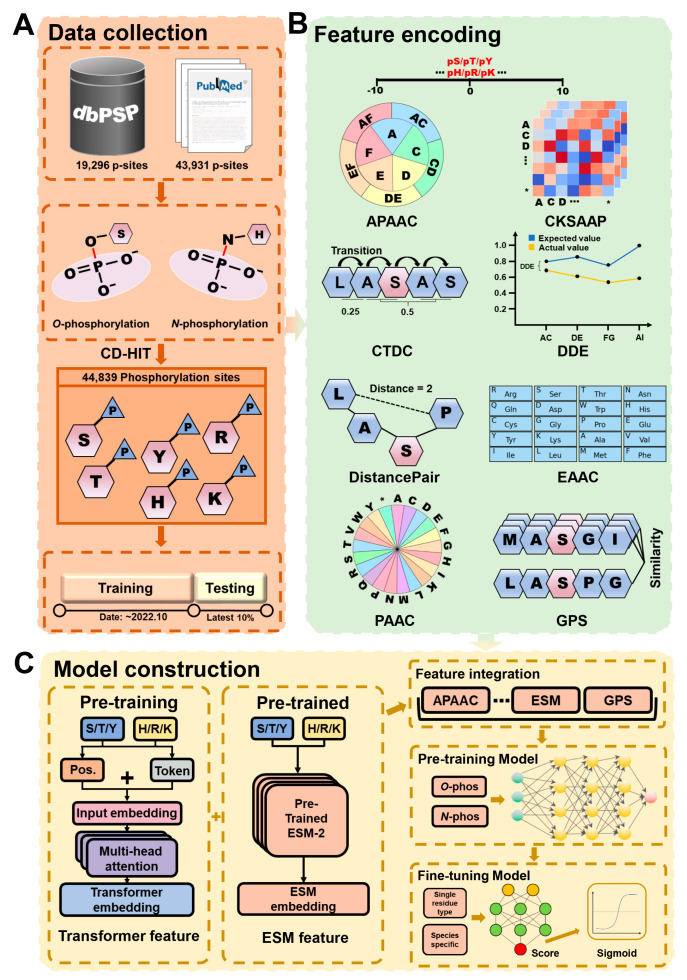
The procedure for the development of the GPS-pPLM, including data collection, feature encoding, and model construction. (**A**) Data preparation of p-sites curated from the literature and dbPSP 2.0. (**B**) Sequence feature encoding of the GPS-pPLM algorithm. In total, 8 types of sequence features were used, including APAAC, CKSAAP, CTDC, DDE, distance pair, EAAC, PAAC, and GPS. (**C**) Model construction methods used for GPS-pPLM. Two machine learning approaches, including transformer and DNN, were used for the construction of predictive models. We pretrained two general models, the *O*-phosphorylation model and the *N*-phosphorylation model, and then the pretrained models were fine-tuned using data specific to a single-residue type or species.

First, we obtained 19,296 experimentally identified p-sites in 8586 proteins for prokaryotes from dbPSP 2.0 [23]. To avoid missing any data, we utilized a series of keywords, including “bacteria phosphoproteomics”, “archaea phosphorylation”, “archaebacterial phosphorylation”, and “prokaryotic phosphorylation”, to additionally collect 43,931 experimentally identified p-sites from the PubMed literature. We merged the two datasets and obtained 50,323 nonredundant p-sites in 18,539 proteins. A widely used clustering program, CD-HIT [41], was adopted to clear homologous sites with a threshold of 40% sequence similarity. If two phosphorylated proteins are modified at the same positions with >40% sequence identity, only one of them is reserved (Figure 1A). In total, we obtained 44,839 nonhomologous p-sites in 16,041 proteins from 95 prokaryotes. This benchmark dataset was used for further training and testing.

We then defined a p-site peptide PSP(*n*, *n*) as a phosphorylation residue flanked by *n* residues upstream and *n* residues downstream. We adopted PSP(10, 10) to enable rapid training. For each phosphorylatable residue type, the PSP(10, 10) items surrounding experimentally identified p-sites were considered positive data, whereas those surrounding nonphosphorylated sites of the same protein were considered negative data. For residues located near the N- or C-terminus of the protein sequences, one or multiple characters “*” were added to complement the PSP(10, 10) items. In addition, we used PSI-CD-HIT [41] to clear homologous sites with a stringent threshold of 25% sequence similarity.

### 2.2. Performance Evaluation Measurements

To evaluate the accuracy of the GPS-pPLM, the true-positive (TP), true-negative (TN), false-positive (FP), and false-negative (FN) values were separately calculated for each model. *Sn*, *Sp*, accuracy (*Ac*), and the Mathew correlation coefficient (*MCC*) were subsequently evaluated, as follows:

*Sn* = TP/(TP + FN) (1)

*Sp* = TN/(TN + FP) (2)

*Ac* = (TP + TN)/(TP + FP + TN + FN) (3)

*MCC* = ((TP × TN) − (FN × FP))/√((TP + FN) × (TN + FP) × (TP + FP) × (TN + FN)) (4)

For each model, 4-fold cross-validation was performed to evaluate the accuracy and robustness. To compare the GPS-pPLM with existing tools, we split the benchmark dataset into training and testing datasets via a timestamp-based method. The training dataset comprises 40,348 p-sites collected prior to October 2022 (Supplementary Table S2), whereas the testing dataset consists of data points collected after October 2022 (Supplementary Table S3), including 4491 p-sites used for independent evaluation. The receiver operating characteristic (ROC) curve was generated on the basis of the *Sn* and 1 − *Sp* scores, and the area under the curve (AUC) was calculated (Supplementary Table S4).

### 2.3. Feature Encoding

First, each PSP(10, 10) is encoded by 8 types of sequence features, including amphiphilic pseudo-amino acid composition (APAAC), composition of *k*-spaced amino acid pairs (CKSAAP), composition, transition, and distribution of composition (CTDC), dipeptide deviation from the expected mean (DDE), pseudo-amino acid composition of distance pairs and reduced alphabet (DistancePair), enhanced amino acid composition (EAAC), and pseudo-amino acid composition (PAAC), respectively [42], as well as the peptide similarity encoded by the group-based prediction system (GPS) method [43] (Figure 1B). We also considered two types of contextual features (Figure 1C). One contextual feature was derived from a transformer-based model trained with our own training dataset. Due to the limitation of currently available p-sites in prokaryotes, the contextual information might not be comprehensively captured. Thus, we further included an additional contextual feature derived from a large PLM, esm2_t33_650M_UR50D.pt (https://github.com/facebookresearch/esm, accessed on 6 November 2024), which was pretrained using 250 million protein sequences [29]. From each model, the output of the last hidden layer was retrieved as contextual features for further model training.

The transformer-based model architecture consists of an embedding module and a transformer encoder module. In the embedding module, PSP(10, 10) is encoded as a position feature and token feature, and the final feature E of PSP(10, 10) is defined as follows:

E = Embedding_token + Embedding_pos(5)

The transformer encoder module is composed of a multihead attention mechanism and a feedforward neural network. The multihead attention mechanism consists of 4 individual heads stacked together. For each head, the input feature *E* is multiplied by three linear layers *Wq*, *Wk*, and *Wv* to obtain the following three matrices: Query (*Q*), key (*K*), and value (*V*). The attention mechanism can be calculated as follows:

Attention(Q, K, V) = softmax((QK^T^)/√(d_k_))V (6)

Then, the attention heads are concatenated, and the final attention layer is represented as follows:

MultiHead(Q, K, V) = Concat(head_1_, …, head_4_)Wo (7)

where *Wq*, *Wk*, *Wv*, and *Wo* are the weight matrices that need to be trained, and *d_k_* represents the dimension of *K*, while *K^T^* represents the transpose of *K*.

The output of the multihead attention layer is processed by the normalization layer and enters the feed-forward network layer (*FFN*). The *FFN* consists of two linear transformations with rectified linear unit (*ReLU*) activation between them. The *ReLU* activation function is defined as follows:

ReLU(x) = max(0, x)(8)

The final scores from the output layer were calculated via the sigmoid function to evaluate the probability of the PSP(10, 10) item being a p-site. The sigmoid score ranged from 0 to 1, and a higher sigmoid score represents a higher probability of the PSP(10, 10) item being a true p-site. The sigmoid function is defined as follows:

sigmoid(x) = 1/(1 + e^(−x))(9)

### 2.4. Algorithm of the GPS-pPLM

The framework of the GPS-pPLM is shown in Figure 1C. We pretrained two general models, including the *O*-phosphorylation model and the *N*-phosphorylation model. The *O*-phosphorylation model was trained using pS, pT, and pY sites (*O*-phosphorylation dataset), and, similarly, the *N*-phosphorylation model was constructed using phosphohistidine (pH), phosphoarginine (pR), and phospholysine (pK) sites (*N*-phosphorylation dataset). Specifically, each PSP(10, 10) within the *O*-phosphorylation dataset and *N*-phosphorylation dataset was encoded into a set of 10 features. The features were used to train 10 separate DNN models, each of which can be used to score PSP(10, 10). Thus, each PSP(10, 10) peptide can be re-encoded as a 10-dimensional number vector, as follows:

V = (D1, D2, D3, …, D10)(10)

The vector *V* was used to train the *O*-phosphorylation model and *N*-phosphorylation model, whereas 4-fold cross-validations were carried out to evaluate the performance.

Next, the pretrained models were fine-tuned via transfer learning to construct predictive models for single-residue types, as well as prokaryotic species. In brief, we used the parameters of the pretrained 10-feature models of the *O*-phosphorylation dataset as the initial parameters of the predictors of pS, pT, or pY. During predictor training, the parameters of the hidden layers were frozen, and the parameters of the input and output layers were updated. Each fine-tuned DNN model of 10 features was finally integrated to calculate a predicted score for the evaluation of the possibility of modified residues. Using a similar strategy, we ultimately constructed 6 residue types and 134 species-specific predictors.

Both the pretrained and fine-tuned DNN models consisted of a seven-layer architecture, comprising an input layer, five hidden layers, and an output layer, with ReLU activation functions applied. All of the models were implemented via PyTorch 1.10.0 and trained on a computer equipped with an NVIDIA GeForce GTX 1060 GPU, an Intel(R) Core™ i5-8400K @ 2.80 GHz central processing unit (CPU), and 32 GB of RAM.

### 2.5. Integrated Annotations

We integrated several tools to annotate our prediction results on the website. IceLogo, a widely used tool for visualizing web logos (https://iomics.ugent.be/icelogoserver/, accessed on 6 November 2024) [44], was employed to display enriched and depleted amino acids. All positive PSP(10, 10) items were uploaded to IceLogo, generating sequence logos for single-residue p-sites. Additionally, 3Dmol.js [45] was utilized to visualize the three-dimensional structure and predicted sites of the proteins. Protein disorder propensity values were assessed via IUPred [46]. The accessible surface area (ASA) and secondary structure were determined via NetSurfP [47]. Furthermore, in addition to basic statistical analyses, knowledge from 22 public resources was incorporated.

### 2.6. The Hypergeometric Test

Hypergeometric tests were adopted for motif enrichment analysis of the training dataset, and we defined the following:

*N* = The number of PSP(10, 10) items;

*n* = The number of PSP(10, 10) items that contain motifs;

*M* = The number of positive PSP(10, 10) items;

*m* = the number of positive PSP(10, 10) items that contain motifs.

Then, the enrichment ratio (E-ratio) was calculated, and the *p*-value was calculated with the hypergeometric distribution as follows:

E-ratio = (*m*/*M*)/(*n*/*N*) (11)

*p*-value = ∑_(*m’* = *m*)^*n* (C(*M*, *m’*) × C(*N* − *M*, *n* − *m’*))/C(*N*, *n*), (E-ratio > 1) (12)

### 2.7. Model Interpretation and Attention-Mechanism-Based Motif Analysis

We employed deep Shapley Additive exPlanations (SHAP) (https://github.com/shap/shap, accessed on 6 November 2024) [48] to analyze feature contributions in the GPS-pPLM. For each single-residue type model, deep SHAP analysis was conducted for each position of the PSP(10, 10) items. To quantify the contribution of each feature to the model via SHAP values, the absolute values of the SHAP values for each feature were first calculated. The summed score of each feature represents its final contribution to the construction of the predictive model.

In the GPS-pPLM, the attention layers of the transformer-based model are adopted to measure and extract attention weight through the multihead attention mechanism. The model consists of three block layers with 4 heads each, resulting in 12 attention matrices for each PSP(10, 10) item. Initially, attention matrices from all positive PSP(10, 10) items were calculated and normalized to generate position-specific attention weight matrices. These matrices were visualized as heatmaps via the Python package Matplotlib [49]. Additionally, we evaluated the attention weights between the modified residues and other amino acids across various positions and obtained attention weight matrices for amino acid types and their positions, which were used for further motif analyses.

### 2.8. Implementation of the Web Service

The online service of the GPS-pPLM was constructed with PHP 7.4 and JQuery 1.4.4, and the back end was written in Python 3.8. The residue type and species can be selected, and 3 threshold options, “High”, “Medium”, and “Low”, can be chosen with *Sp* values of ~95%, ~90%, and ~85%, respectively. We also implemented an “All” option to allow for the predictions of all the results to be shown. The GPS-pPLM has been extensively tested on various web browsers, including Internet Explorer, Mozilla Firefox, and Google Chrome, to provide a robust and freely available service at: https://pplm.biocuckoo.cn/, accessed on 6 November 2024.

### 2.9. Analysis of Hypervariable Regions and Co-Evolving Regions

From a widely used database, OrthoDB (https://www.orthodb.org/, accessed on 6 November 2024) [50], we downloaded 3638 orthologous groups of *E. coli* proteins at the Enterobacteriaceae level, as well as 2350 orthologous groups of *Staphylococcus aureus* proteins at the Staphylococcaceae level. For each orthologous group, we performed multiple sequence alignment (MSA) using MUSCLE [51]. To determine the hypervariable regions in *E. coli* protein sequences, we used the Protein Variability Server (PVS, http://imed.med.ucm.es/PVS/, accessed on 6 November 2024) [52] to analyze the corresponding MSA file for each orthologous group. In the default configuration of PVS, the Shannon method was used, and regions with a Shannon score of > 1 were calculated as hypervariable regions. For the analysis of co-evolving regions, we used a mainstream Python package called pydca, which was implemented in direct coupling analysis (DCA) to infer co-evolving pairs of amino acid residues from MSA files [53,54,55]. As previously described [54], the top 20 amino acid pairs with the highest DCA scores were taken as co-evolving residue pairs for each protein. Next, we used the GPS-pPLM to predict p-sites in proteins of *E. coli* and *S. aureus*, with a high threshold. Then, the hypergeometric test was used to analyze whether p-sites are more likely to occur in hypervariable regions or co-evolving regions.

## 3. Results

### 3.1. Performance Evaluation and Comparison

**Figure 2:**
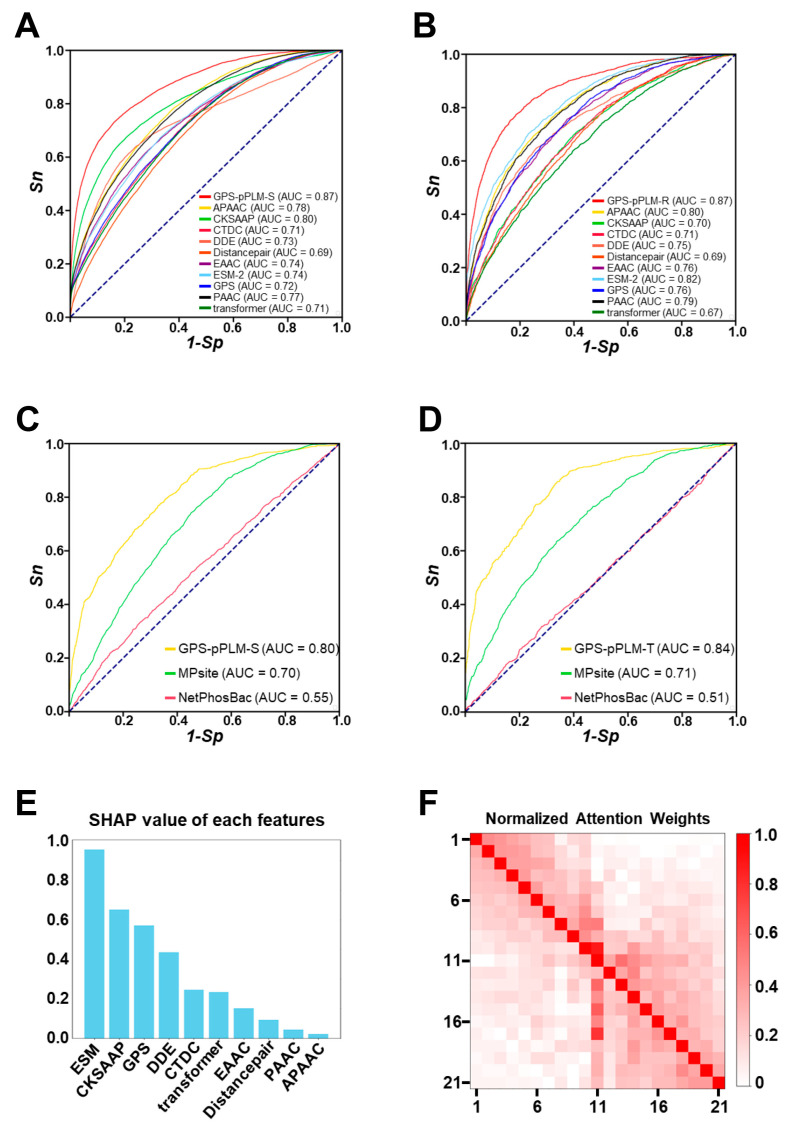
Performance evaluation and comparison of the GPS-pPLM. (**A**,**B**) Performance evaluation of the pS and pR predictors. The accuracies of the 10-feature models and integrated models were evaluated via a 4-fold cross-validation method. (**C**,**D**) Performance comparison between the GPS-pPLM and other existing predictors on the S p-site and T p-site independent testing data, including MPsite and NetPhosBac. (**E**) Evaluation of 10 types of features contributing to the *O*-phosphorylation model by measuring the SHAP score for each feature. (**F**) Visualization of normalized attention weights for the relationships between different positions in S/T p-sites.

To evaluate the accuracy of our prediction models, 4-fold cross-validation was employed, and the AUC value was calculated for each of the 10-feature models specific for two general models and six types of amino acid residues, as well as for the final integrated models. For the *O*-phosphorylation general model, the AUC value was 0.77, while the AUC value for the *N*-phosphorylation general model was 0.71, with the homology filtered at the 40% threshold (Supplementary Figure S1A,B). Using the 50,323 p-sites without homologous filtration or with a stringent threshold of 25%, we found that the performance values of the two general models were not significantly changed (Supplementary Figure S1C,D). For the prediction of pS sites, the AUC values of the predictive models of single features ranged from 0.69 to 0.80, and the AUC value of the trained model integrating 10 features was 0.87 (Figure 2A). This result demonstrated that the AUC value of the integrated model increased by 8.8–26.1%, compared with that of the other models, when a single feature was used. Similarly, the AUC values of the pR predictors trained with a single feature ranged from 0.67 to 0.82, and the AUC value of the integrated model reached 0.87, indicating a 6.1–29.9% increase in the AUC values compared with those of the other models trained with a single feature (Figure 2B). For the remaining four types of phosphorylated residues, the AUC values of the integrated models ranged from 0.79 to 0.91, and all of the integrated models presented higher AUC values than the pretrained models did (Supplementary Figure S1E–H). To evaluate our models against accessible existing tools, we selected 4492 p-sites identified after October 2022 as independent testing datasets via a timestamp method. Next, we selected two available tools, MPsite and NetPhosBac, for performance evaluation. For these tools, which provide only the prediction of pS sites and pT sites, our evaluations focused on the performance of pS- and pT-specific predictors via our independent validation set. The results indicated that the AUC values of the pS predictor in GPS-pPLM were 14.3% and 45.5% greater than those of MPsite and NetPhosBac, respectively (Figure 2C). Similarly, for the pT predictor, the AUC values of the GPS-pPLM increased by 18.3% and 64.7%, respectively (Figure 2D).

Next, to investigate the contributions of 10 features in the GPS-pPLM, a widely used method, SHAP, was employed for the evaluation of our developed model. Our analysis demonstrated that all 10 features exhibited various contributions to the construction of our predictive model (Figure 2E and Figure S1I). In particular, we observed that the contextual features from the pretrained PLM and ESM had the highest SHAP scores, indicating the importance of contextual information embedded in protein sequences for the prediction of modification sites. Moreover, the sequence feature encoded by the GPS method obtained a high score (Figure 2E and Figure S1I), indicating that sequence similarity is essential for inferring prokaryotic p-sties. The contextual information encoded by the transformer architecture was also useful for the development of the GPS-pPLM (Figure 2E and Figure S1I). In addition, we trained models only using contextual features, encoded by the transformer, pretrained ESM, or both. Compared to models using all features, the AUC value for the *O*-phosphorylation model decreased from 0.77 to 0.72, and the AUC value for *N*-phosphorylation model reduced from 0.71 to 0.68, indicating that both sequence features and contextual features were informative for the prediction of prokaryotic p-sites (Supplementary Figure S1J,K). In the transformer-based pretrained model, to further explore the relationships between amino acids at various positions in PSP(10, 10) items, an attention mechanism was employed to analyze the in-context information of protein peptides. The results indicate that each amino acid showed the highest attention to itself, and the attention from position *i* to position *j* was usually not equal to the attention from position *j* to position *i*. The residues in the vicinity of <six amino acids also received high attention scores (Figure 2F and Figure S1L). Taken together, combining two deep learning methods and 10 features facilitates the establishment of a high-accuracy GPS-pPLM.

### 3.2. Motif Analysis of Known Prokaryotic p-Sites

**Figure 3:**
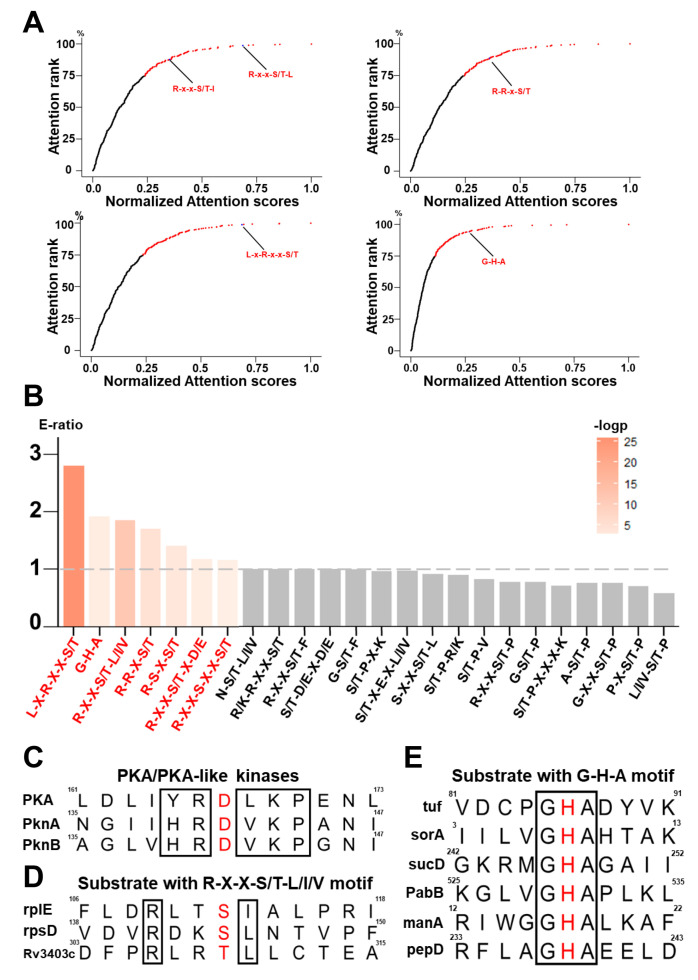
Identification of prokaryotic p-site motifs. (**A**) Eukaryotic-like phosphorylation motifs identified in prokaryotes through the attention mechanism, including R-X-X-S/T-L/I/V, R-R-X-S/T, L-X-R-X-X-S/T, and G-H-A. (**B**) The enrichment results of 24 motifs containing two or more amino acids were obtained from all training data (*p*-value < 0.01). These motifs were identified via the motif-x tool from the phosphoproteomic data of 48 eukaryotes. (**C**) Sequence alignment results of the PKA, PknA, and PknB kinase functional sites. (**D**) The substrates that can be specifically phosphorylated by PknB include R-X-X-S/T-L/I/V motifs in prokaryotes. (**E**) Display of sequences near the p-site of the G-H-A motif in the training dataset.

We further analyzed the correlation between amino acid pairs at different positions in the PSP(10, 10) items. For positive PSP(10, 10), we calculated the attention weights between modified residues and the other amino acids at various positions to evaluate amino acid preference at each position (Supplementary Figure S2A,B). For the *n* positions in PSP(10, 10), 20^n^ types of amino acids were present. We summed the attention weights of amino acids at each position and employed min–max normalization to process the scores ranging from 0 to 1. This score represents the likelihood of the composition being a potential sequence motif, and a higher score indicates a greater probability. We analyzed the amino acid preferences of pS/pT (−3,1) (the third upstream and first downstream positions of pS/pT), pS/pT (−3, −2), pS/pT (−5, −3), and pH (−1,1), and the analysis results indicated that several well-characterized eukaryotic-like phosphorylation motifs in prokaryotes were recognized by the attention mechanism in the training of predictive models (Figure 3A).

According to previous studies, 29 protein motifs were identified from phosphoproteomic data of 48 eukaryotes via the motif-x tool [56]. Among these, we selected 24 motifs that contained two or more amino acids as benchmark datasets for enrichment analysis. On the basis of our dataset of prokaryotic p-sites, we utilized the hypergeometric test method to perform an enrichment analysis of sequence motifs. The results indicated that several of the sequence motifs identified were enriched in both eukaryotes and prokaryotes (Figure 3B). To further confirm the essential role of these enriched motifs in prokaryotes, we reviewed the existing knowledge regarding the important kinase-specific p-sites in prokaryotes. In prokaryotic cells, some protein kinases are homologous to eukaryotic protein kinases and are currently termed ELKs. For example, the PknB gene in *Mycobacterium tuberculosis* is structurally similar to mouse cAMP-dependent protein kinase (PKA) in the activated state [57] (Figure 3C). The substrates of PKA typically contain R-X-X-S/T-L/I/V motifs in eukaryotes, and it has been experimentally confirmed that PknB can also specifically phosphorylate substrates containing these sequence motifs in prokaryotes [58] (Figure 3D). In addition, one of the existing 3-pHis mAbs, SC44, exhibits pseudosequence specificity and preferentially recognizes Gly-3pHis-Ala motifs, which are identified in the active sites of ACLY and SCSa [3]. Consistent with this finding, such a motif was also found in our datasets (Figure 3E). Overall, our analysis results indicated that the attention mechanism of the transformer-based model captured amino acid preferences at specific positions and extracted the characteristics of the motifs that underwent phosphorylation.

### 3.3. Evaluation of Species-Specific Predictive Models

**Figure 4:**
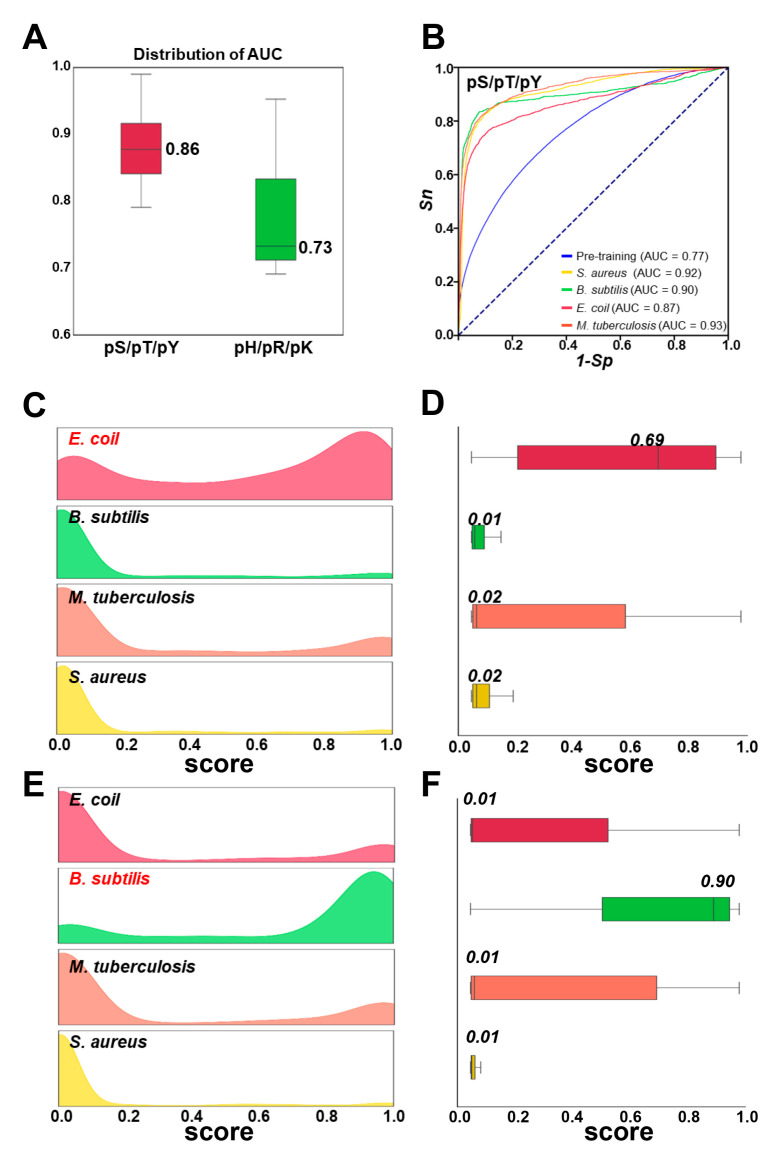
Evaluation of the prediction results of different species-specific predictors. (**A**) Distribution of AUC values for 95 species-specific predictors in the *O*-phosphorylation dataset and 39 species-specific predictors in the *N*-phosphorylation dataset. (**B**) AUC values of four extensively studied prokaryotic species predictors, namely, *B. subtilis*, *E. coli*, *M. tuberculosis*, and *S. aureus*. (**C**,**D**) The distribution of prediction scores for *E.-coli*-positive data using four species-specific predictors. (**E**,**F**) The distribution of prediction scores for *B.-subtilis*-positive data using four species-specific predictors.

In addition to the predictors for the six phosphorylatable residue types, we also constructed species-specific predictors via transfer learning to fine-tune the pretrained models. The *O*-phosphorylation and *N*-phosphorylation datasets were categorized into single-species datasets on the basis of distinct species. For each species, the dataset containing at least four positive samples was used to train species-specific p-site predictors. Ultimately, we obtained 95 species-specific predictors for the inference of *O*-phosphorylation and 39 species-specific predictors for *N*-phosphorylation site prediction. The AUC values for each model were calculated and are presented in Figure 4A. Because the number of experimentally identified *O*-phosphorylation sites in prokaryotic cells is greater than the number of *N*-phosphorylation sites, the median AUC value for the predictors in the *O*-phosphorylation dataset was 0.88, and the median AUC value of the predictive models in the *N*-phosphorylation dataset was 0.73. For our analysis, we selected four extensively studied prokaryotic species from the *O*-phosphorylation dataset, namely, *Bacillus subtilis*, *E. coli*, *M. tuberculosis*, and *S. aureus*, to further analyze the specificity of the species-specific predictors. The AUC values for these four species-specific predictors ranged from 0.87 to 0.93, which were higher than the AUC value of 0.77 in the general model for the prediction of *O*-phosphorylation sites, supporting the improved performance of the predictive models after fine-tuning (Figure 4B).

To further analyze the specificity of the species-specific predictors, we evaluated the performance across datasets from various species. The *E.-coli*-specific model was used to evaluate the training datasets of *B. subtilis*, *E. coli*, *M. tuberculosis*, and *S. aureus*. The results demonstrated that the *E.-coli*-specific model was able to effectively distinguish the positive and negative datasets from the *E. coli* dataset*,* and the datasets separately received high and low scores. For the other three species, datasets with low predictive scores were obtained (Figure 4C). Next, we scored the positive samples from each of the four datasets and presented the results (Figure 4D). The *E.-coli*-specific model yielded high scores for the positive datasets from *E. coli* and had a median score of 0.69, whereas the positive samples from the other datasets received lower scores. A similar analysis was performed using the *B.-subtilis*-specific model. The results indicated that the *B.-subtilis*-specific model could effectively distinguish *B. subtilis*, assigning high scores to positive samples with a median of 0.90 (Figure 4F). In the prediction of the other three species, the predictive scores were dramatically lower (Figure 4E). Our findings suggest that the sequence characteristics surrounding p-sites in various prokaryotic species are different. In summary, our analysis revealed that the species-specific predictors possess more specialized predictive capabilities for individual species.

In addition, we computationally determined hypervariable regions and co-evolving regions in protein sequences of *E. coli* and *S. aureus*, using PVS [52] and pydca [54], respectively. From the MSA files of 3638 orthologous groups of *E. coli* proteins, we identified 385,508 residues in hypervariable regions and 72,760 pairs of co-evolving residues. For 2350 orthologous groups of *S. aureus*, we identified 385,712 hypervariable residues and 47,000 pairs of co-evolving residues. Using the hypergeometric test, it was found that the predicted p-sites were more likely to occur in both hypervariable regions and co-evolving regions (Supplementary Figure S3A,B, *p*-value < 1.91 × 10^−17^).

### 3.4. Usage of the GPS-pPLM Web Server

**Figure 5:**
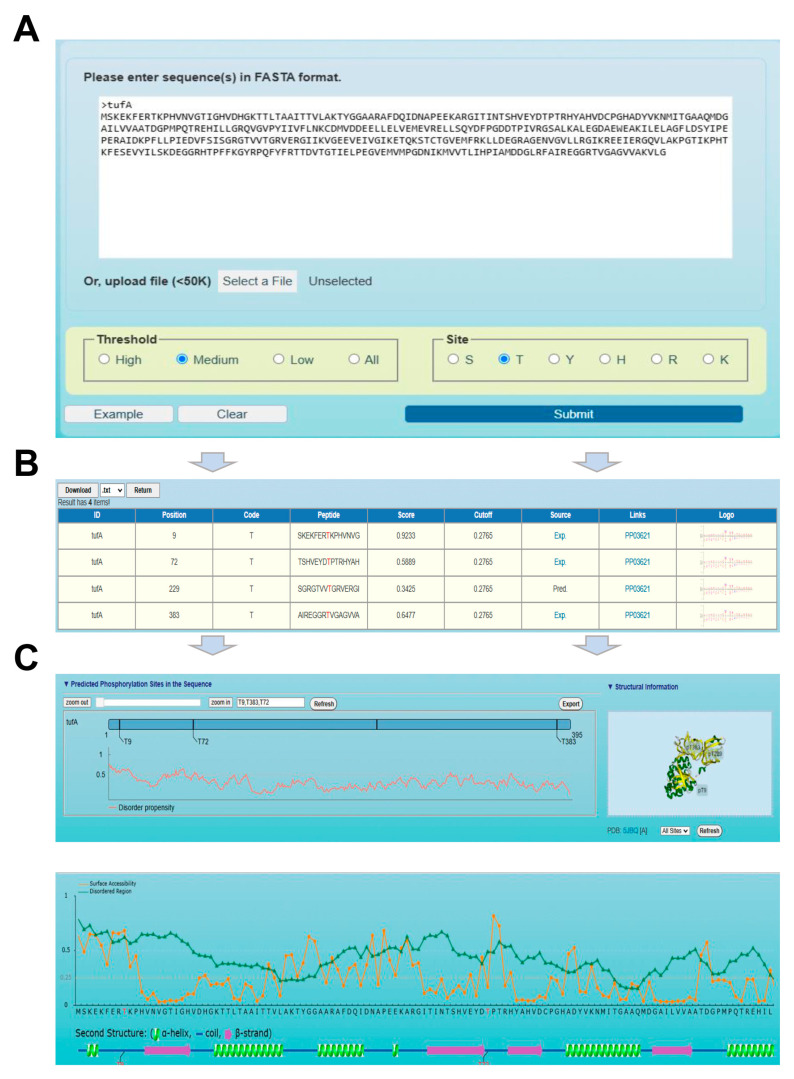
Usage of the GPS-pPLM web server. (**A**) The interface of sequence submission. Users can input protein sequences in FASTA format or enter UniProt accession numbers and select 3 different thresholds and 6 different residue types to predict p-sites. (**B**) Presentation of the prediction results of the example. In the tabular list, the predictive results include the position of the p-site, residue type, prediction score, cut-off value, identification via experimental or computational methods, and links to dbPSP 2.0. (**C**) Comprehensive annotations of the prediction results. The line chart shows the disorder score of the p-sites, and the location of the p-sites is explained in the 3D structure exported from the PDB database. The ASA score and disorder score of the p-sites are also calculated in the comprehensive mode.

For convenience, we developed a user-friendly web server, GPS-pPLM (https://pplm.biocuckoo.cn, accessed on 6 November 2024). In the GPS-pPLM tool, we implemented three distinct types of predictors, including the single-residue predictor, the species-specific predictor, and a comprehensive predictor that incorporates additional annotations such as secondary structure and surface accessibility. For the prediction of p-sites, one or several protein sequences in FASTA format or UniProt accession numbers can be submitted. In the prediction interfaces, the users first select a resulting filtering threshold from the threshold panel located below the web interface. Moreover, visiting users can choose a site or species according to their usage. After clicking the “Submit” button, users can access the predicted results and additional annotations (Figure 5A). Following a brief processing period, the output for each input sequence can be visualized individually. The “Next Protein” button allows users to retrieve the subsequent protein’s predictions. The predicted results provide detailed information on potential p-sites, including nine types of data, such as “ID”, “Position”, “Code”, “Peptide”, “Score”, “Cut-off”, “Source”, “Links”, and “Logo” (Figure 5B).

In the “Source” column, “Exp” indicates that experimental evidence is available via a PubMed link. The “Link” provides a link from the p-site to the dbPSP 2.0 database [23]. For each residue type, the sequence logos of PSP(10, 10) items in the positive dataset are displayed in the “Logo” column. In the default configuration, the top three p-sites with the highest prediction scores are displayed in a schematic diagram of the protein sequence. Additionally, the disordered propensity score of residues, determined by IUPred [46], is shown as a line chart. If accessible, 3Dmol.js displays the 3D structural information of potential p-sites. In the comprehensive mode, surface accessibility and secondary structures, including alpha helices, beta strands, and coils, are assessed (Figure 5C). All of the predicted results are available for download in .txt or .xlsx formats. The “Export” button adjacent to the images allows users to download.png files. Detailed instructions are provided on the “User Guide” page, providing comprehensive guidance on the usage of the online service and the interpretation of the predictive results.

### 3.5. GPS-pPLM Predicts p-Sites of the E. coli Protein tufA

The GPS-pPLM is a user-friendly web server, and the amino acid sequence of the classical *E. coli* protein tufA (UniProt ID: P0CE47) was chosen as a representative example to demonstrate the use of the GPS-pPLM. The GTPase elongation factor, tufA, is an essential component of the translational machinery in *E. coli*. It plays pivotal roles in aminoacyl-tRNA delivery, translation accuracy, and the regulation of protein synthesis efficiency and adaptability [59,60]. Notably, phosphorylation is a significant regulatory mechanism influencing the function of tufA. Recent studies indicate that the phosphorylation of tufA/EF-Tu impacts its conformational dynamics, eventually leading to the impairment of its function in protein synthesis [61,62,63].

**Figure 6:**
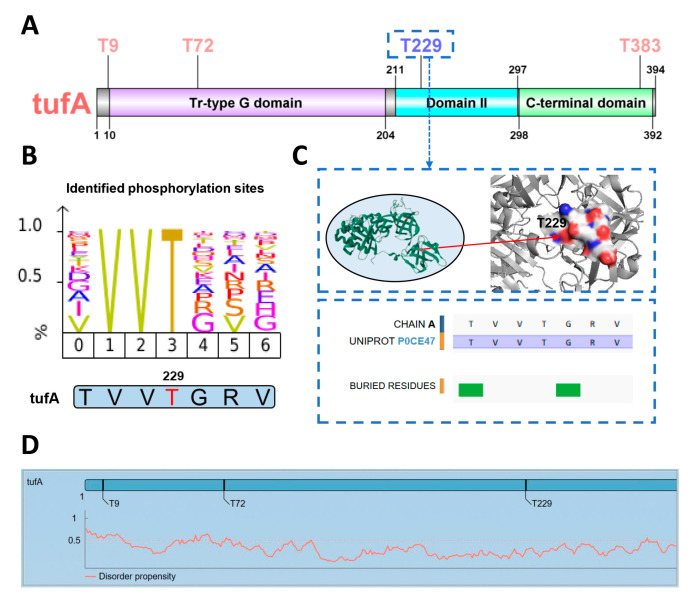
The prediction results for the *E. coli* protein tufA. (**A**) The T residues predicted as p-sites in the tufA protein, including T9, T72, T229, and T383. (**B**) Amino acid frequency of sequences containing VVT motifs at known p-sites and the amino acid sequence near T229. (**C**) The 3D structure of T229 and the annotation results from PDB. (**D**) Annotation of tufA p-sites. The disorder score was measured for the p-sites.

Using the pT predictor of the GPS-pPLM, four pT sites in the tufA protein were computationally analyzed, including T9, T72, T229, and T383 (Figure 6A). Among these, three p-sites, T9, T72, and T383, were experimentally validated [61,63,64]. Previous reports have shown that phosphorylation at the T383 position decelerates the conformational dynamics of tufA/EF-Tu, preventing it from binding aminoacyl-tRNA and, consequently, leading to the inactivation of protein function [61,63,64]. Phosphorylation at T9 and T72 was identified via phosphoproteomic profiling [65,66]. The T72 residue is located within the tr-type G domain, which functions as a molecular switch, regulating translation by alternating between an inactive GDP-bound state and an active GTP-bound state [61]. Phosphorylation at T72 may affect the dynamic behavior of the GTP-bound tr-type G domain, resulting in protein dysfunction.

Using the GPS-pPLM, T229 in the prokaryotic protein tufA was computationally predicted as a p-site that had not been previously recognized. The analyses revealed that this threonine residue was located in the VVT motif, and the VVT motif was previously identified to be phosphorylated in other prokaryotic proteins. Thus, our predictive results suggested that the T229 residue of the tufA protein might undergo phosphorylation (Figure 6B). Furthermore, a zoomed 3D structure shows that T229 is presented as an exposed residue, and PDB [67] annotations demonstrate that the V, V, and T residues are not buried residues, suggesting that T229 might be recognized by catalytic enzymes (Figure 6C). Moreover, it was reported that mutations in the adjacent R231 residue result in a pulvomycin-resistant strain, suggesting that phosphorylation at T229 might influence *E. coli* antibiotic persistence [68]. Additionally, other evidence has demonstrated that EF-Tu mutants, including those at T229, can increase the binding affinity of Sep-tRNA [69,70]. On the basis of this information, T229 might be phosphorylated, and this modification site might influence the interaction with other functional molecules in the *E. coli* strain. In addition, the GPS-pPLM provided additional annotations for the analyzed modification sites, including disorder scores, zoomed 3D structures, and experimental validation information from dbPSP 2.0 [23] (Figure 6C,D and Supplementary Table S5). Taken together, the GPS-pPLM could be used for the prediction of prokaryotic p-sites with well-annotated information, which is helpful for the study of phosphorylation in prokaryotes.

## 4. Discussion

Protein phosphorylation is a critical posttranslational modification in prokaryotes that plays a pivotal role in regulating nearly all biological processes [1,2,3,4]. Phosphorylation events in prokaryotes are closely linked to infection, antibiotic persistence, and virulence [5,13,14]. For example, penicillin-binding protein and serine/threonine kinase-associated (PASTA) kinases significantly contribute to the virulence and beta-lactam resistance of several key pathogens by modulating metabolism, cell division, and cell wall homeostasis [14]. Therefore, identifying new p-sites can enhance our understanding of prokaryotic regulatory mechanisms and provide valuable targets for antimicrobial drug development. With the advancement of sequencing technology, MS-based proteomics techniques have been widely used for the high-throughput identification of p-sites, leading to the emergence of various databases, such as dbPSP 2.0 [23]. On the basis of these data resources, eight p-site prediction tools have been developed, promoting the rapid identification of potential prokaryotic p-sites (Supplementary Table S1).

In this work, we combined eight sequence-based features, as well as two contextual features, applied pretrained methods followed by transfer learning methods, and developed a new prokaryotic p-site prediction tool, GPS-pPLM. We completed a benchmark dataset containing 44,839 known prokaryotic p-sites, with 90% of the data being used for the training dataset and the remaining 10% being used as an independent test dataset according to timestamps. Using 4-fold cross-validation, the pretrained models for *N*-phosphorylation and *O*-phosphorylation presented AUC values of 0.71 and 0.77, respectively (Supplementary Figure S1A,B). The pretrained model was subsequently fine-tuned using data specific to a single-residue type, resulting in six single-residue type predictors. The AUC values of the fine-tuned model ranged from 0.79 to 0.91 (Figure 2A,B and Figure S1E–H), which were higher than those of the pretrained model, demonstrating the superiority of the transfer learning methods. Compared with existing tools, the GPS-pPLM has demonstrated highly competitive accuracy in predicting prokaryotic p-sites, with an AUC increase of 10% to 33% (Figure 2C,D). At present, the existing tools focus only on single-residue type predictors, and relatively little research has been conducted on species-specific predictors. To fill this gap, we also trained 134 species-specific predictors. For the *N*-phosphorylation and *O*-phosphorylation datasets, the median AUC values of the species-specific predictors were 0.73 and 0.86, respectively (Figure 4A), and the results revealed that the species-specific predictor can learn sequence differences across different species (Figure 4C–F). We also calculated the AUC values of different phyla (Supplementary Figure S2C). Compared with general predictors, species-specific predictors can provide more specialized predictive ability for individual species. We also provide a user-friendly web service that offers single-residue type predictors and species-specific predictors, as well as a comprehensive predictor that includes annotations for secondary structure and surface accessibility.

Currently, research on prokaryotic phosphorylation is limited, leading to a scarcity of data. In future work, we plan to continuously integrate experimentally identified prokaryotic p-sites, expand the training dataset, and update and maintain the GPS-pPLM tool. Owing to computational limitations, our current model training uses short peptides. In fact, the transformer architecture can effectively capture contextual relationships in longer sequences. Therefore, we aim to explore training with longer peptide sequences in future iterations. In particular, protein 3D structure determines the function of the protein. Thus, it can be expected that incorporating protein 3D structural features will further improve the prediction accuracy. However, the number of currently available 3D structures is quite limited for prokaryotic proteins, and computational prediction of 3D structures using AlphaFold [71] is time-consuming. Thus, in the current release of the GPS-pPLM, the 3D structural information was provided as an annotation, under the comprehensive mode. As research on prokaryotic phosphoproteomics continues to generate an increasing amount of omics data, we plan to integrate these features into our algorithm. In summary, the GPS-pPLM holds promise for identifying novel prokaryotic p-sites and analyzing phosphorylation regulation in prokaryotes.

## 5. Conclusions

In summary, we compiled a comprehensive dataset of 44,839 nonredundant and nonhomologous p-sites across 16,041 proteins from 95 prokaryotic species. Based on this benchmark dataset, we developed a language model, the GPS-pPLM, for predicting prokaryotic phosphorylation events on six different types of amino acid residues. Both transformer and DNN were used, and 10 types of sequence and contextual features were taken for encoding prokaryotic p-sites. Using transfer learning, we further fine-tuned our general prediction models into 134 species-specific predictors, offering tailored predictive capabilities for individual species. In addition, we applied a multihead attention mechanism to analyze the phosphorylation motifs in prokaryotes. Taken together, we anticipate that the GPS-pPLM can serve as a useful tool for better understanding the mechanisms of prokaryotic phosphorylation.

## Data Availability

All data utilized in this study are provided in the Supplementary Tables, as detailed in the Methods section. The source code of the GPS-pPLM has been uploaded to GitHub (https://github.com/BioCUCKOO/GPS-pPLM, accessed on 6 November 2024).

## Supplementary Materials

The following supporting information can be downloaded at: https://www.mdpi.com/article/10.3390/cells13221854/s1, Figure S1: Additional performance evaluation of the GPS-pPLM. (A,B) Performance evaluation of the pretrained models for *N*-phosphorylation and *O*-phosphorylation via the 4-fold cross-validation method with a 40% threshold. (C,D) Performance comparison of the *N*-phosphorylation and *O*-phosphorylation pretrained models using a 4-fold cross-validation method with CD-HIT thresholds of 25%, 40%, and all p-sites. (E–H) Performance evaluation of the pT, pY, pH, and pK predictors. The accuracies of the 10-feature models and integrated models were evaluated via a 4-fold cross-validation method. (I) Evaluation of 10 types of features contributing to the *N*-phosphorylation model by measuring the SHAP score for each feature. (J) Performance evaluation of the *O*-phosphorylation pretrained model using all features or only contextual features, respectively. (K) Performance evaluation of the *N*-phosphorylation pretrained model using all features or only contextual features, respectively. (L) Visualization of normalized attention weights for the relationships between different positions in H p-sites; Figure S2: Analysis of attention mechanisms and performance of species-specific models. (A,B) Visualization of attention to different positions in the PSP(10, 10) items for 20 amino acids. The visualization results of the S/T p-sites are displayed on the left, whereas the visualization results of the H p-sites are displayed on the right. (C) Distribution of AUC values for different phyla, including *Actinobacteria*, *Bacteroidetes*, *Crenarchaeota*, *Cyanobacteria*, *Deinococcus-Thermus*, *Euryarchaeota*, *Firmicutes*, *Proteobacteria*, *Spirochaetes,* and *Tenericutes*; Figure S3: Analysis of the correlation between phosphorylation sites and hypervariable regions, as well as co-evolving regions. (A) Analysis of the correlation between *O*-phosphorylation sites and hypervariable residues, as well as co-evolving residue pairs in *E. coli* and *S. aureus*. (B) Analysis of the correlation between *N*-phosphorylation sites and hypervariable residues, as well as co-evolving residue pairs in *E. coli* and *S. aureus*. Table S1: Known tools for predicting prokaryotic p-sites; Table S2: The benchmark dataset for training models of the GPS-pPLM; Table S3: The timestamp-based testing dataset; Table S4: The performance of the GPS-pPLM; Table S5: Public resources used in this study.
