# Supplementary material for: GPS-pPLM: A Language Model for Prediction of Prokaryotic Phosphorylation Sites
Source: Cells. 2024 Nov 8;13(22):1854. doi: 10.3390/cells13221854 (PMC11593113; doi:10.3390/cells13221854)
Supplement: Supplementary file 1 [file cells-13-01854-s001.zip › Supplementary Data.pdf]

Supplementary Data:

GPS-pPLM: a language model for prediction of prokaryotic phosphorylation sites

Chi Zhang[1,†], Dachao Tang[1,†], Cheng Han[1], Yujie Gou[1], Miaomiao Chen[1], Xinhe Huang[1], Dan Liu[1], Miaoying Zhao[1], Leming Xiao[1], Qiang Xiao[2], Di Peng[1,*], Yu Xue[1,*]

[1]Department of Bioinformatics and Systems Biology, MOE Key Laboratory of Molecular Biophysics, Hubei Bioinformatics and Molecular Imaging Key Laboratory, College of Life Science and Technology, Huazhong University of Science and Technology, 430074 Wuhan, Hubei, China

[2]School of Artificial Intelligence and Automation, Huazhong University of Science and Technology, 430074 Wuhan, Hubei, China

*Corresponding authors. Department of Bioinformatics and Systems Biology, MOE Key Laboratory of Molecular Biophysics, Hubei Bioinformatics and Molecular Imaging Key Laboratory, College of Life Science and Technology, Huazhong University of Science and Technology, 430074 Wuhan, Hubei, China. Email: xueyu@hust.edu.cn; Department of Bioinformatics and Systems Biology, MOE Key Laboratory of Molecular Biophysics, Hubei Bioinformatics and Molecular Imaging Key Laboratory, Center for Artificial Intelligence Biology, College of Life Science and Technology, Huazhong University of Science and Technology, 430074 Wuhan, Hubei, China. Email: pengdi@hust.edu.cn.

†Chi Zhang and Dachao Tang Joint authors.

Supplementary Figures

Fig S1.

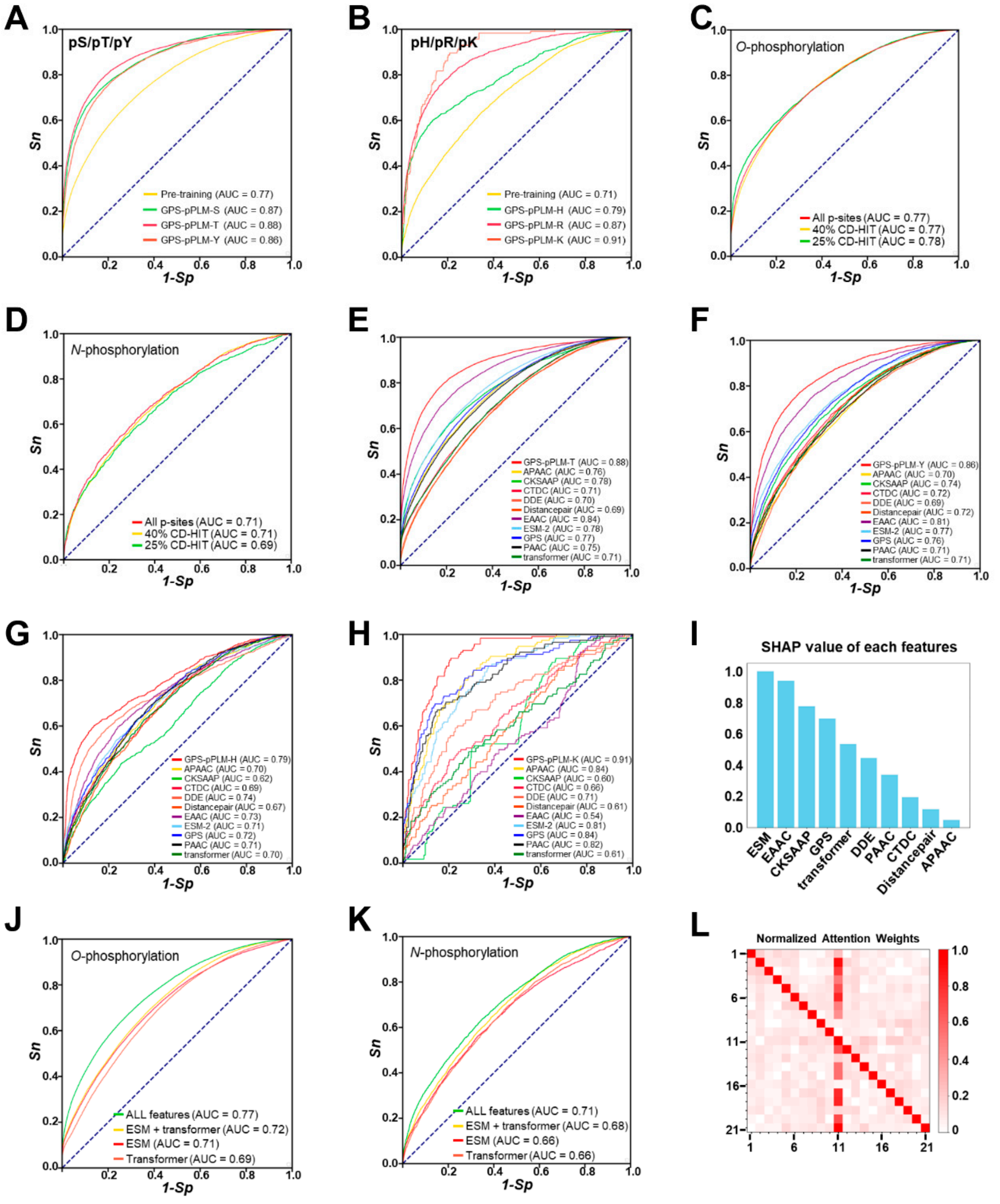

Supplementary Figure S1 – Additional performance evaluation of the GPS-pPLM. (A-B) Performance evaluation of the pretrained models for *N*-phosphorylation and *O*-phosphorylation via the 4-fold cross-validation method with 40% threshold. (C-D) Performance comparison of the *N*-phosphorylation and *O*-phosphorylation pre-trained models using a 4-fold cross-validation method with CD-HIT thresholds of 25%, 40%, and all p-sites. (E-H) Performance evaluation of the pT, pY, pH, and pK predictors. The accuracies of the 10 feature models and integrated models were

evaluated via a 4-fold cross-validation method. (I) Evaluation of 10 types of features contributing to the *N*-phosphorylation model by measuring the SHAP score for each feature. (J) Performance evaluation of the *O*-phosphorylation pre-trained model using all features or only contextual features, respectively. (K) Performance evaluation of the *N*-phosphorylation pre-trained model using all features or only contextual features, respectively. (L) Visualization of normalized attention weights for the relationships between different positions in H p-sites

Fig S2.

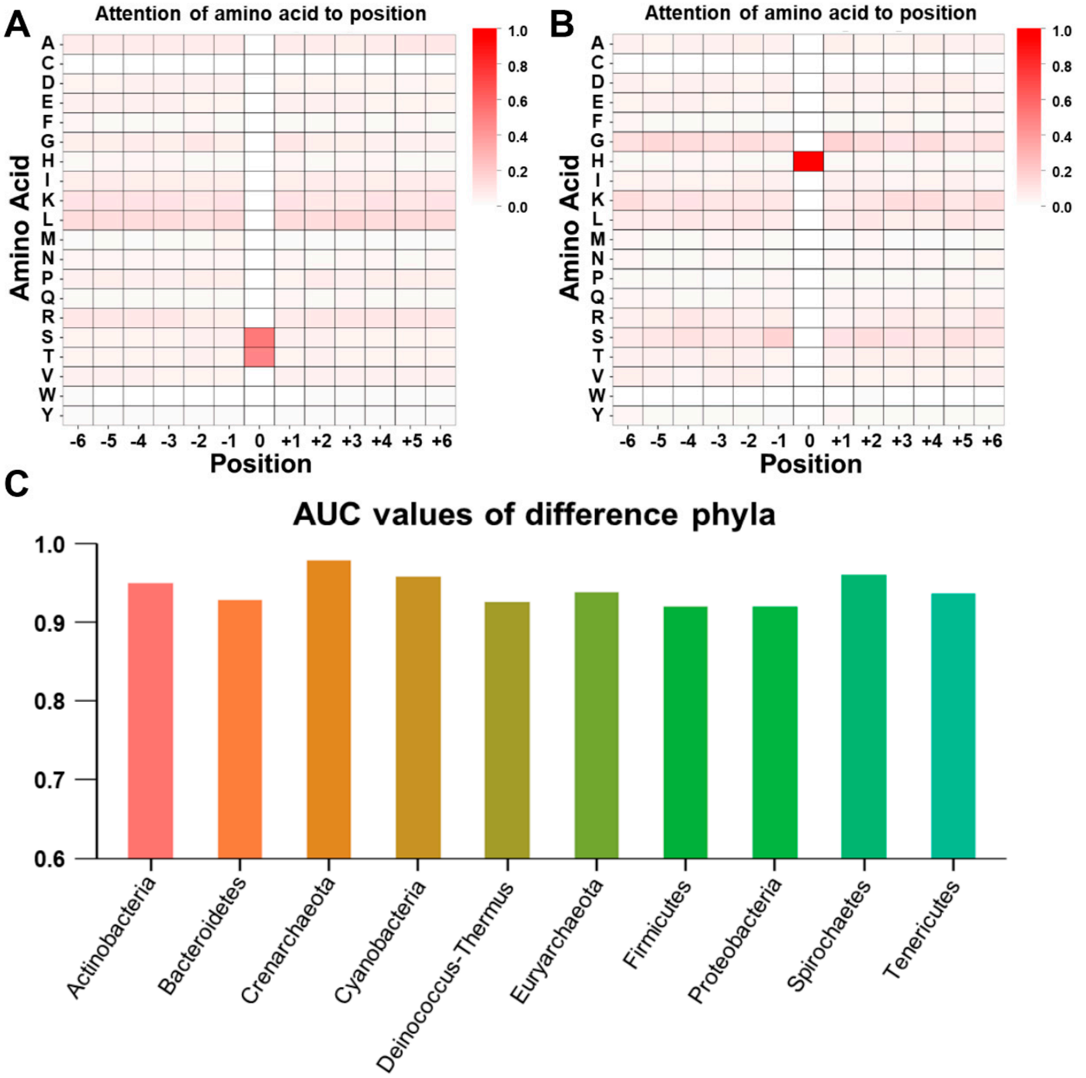

Supplementary Figure S2 – Analysis of attention mechanisms and performance of species-specific models. (A-B) Visualization of attention to different positions in the PSP(10, 10) item for 20 amino acids. The visualization results of the S/T p-sites are displayed on the left, whereas the visualization results of the H p-sites are displayed on the right. (C) Distribution of AUC values for different phyla, including *Actinobacteria*, *Bacteroidetes*, *Crenarchaeota*, *Cyanobacteria*, *Deinococcus-Thermus*, *Euryarchaeota*, *Firmicutes*, *Proteobacteria*, *Spirochaetes,* and *Tenericutes*

Fig S3.

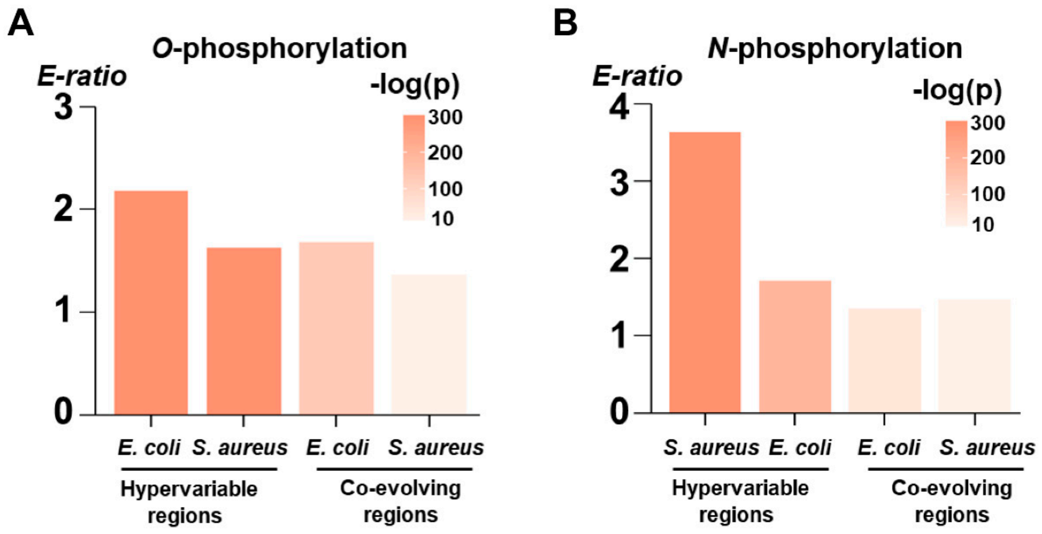

Supplementary Figure S3 – Analysis of the correlation between phosphorylation sites and hypervariable regions as well as co-evolving regions. (A) Analysis of the correlation between *O*-phosphorylation sites and hypervariable residues as well as co-evolving residue pairs in *E. coli* and *S. aureus*. (B) Analysis of the correlation between *N*-phosphorylation sites and hypervariable residues as well as co-evolving residue pairs in *E. coli* and *S. aureus*.
