# Supplementary figures and images for: GPS-pPLM: A Language Model for Prediction of Prokaryotic Phosphorylation Sites
Source: Cells. 2024 Nov 8;13(22):1854. doi: 10.3390/cells13221854 (PMC11593113; doi:10.3390/cells13221854)

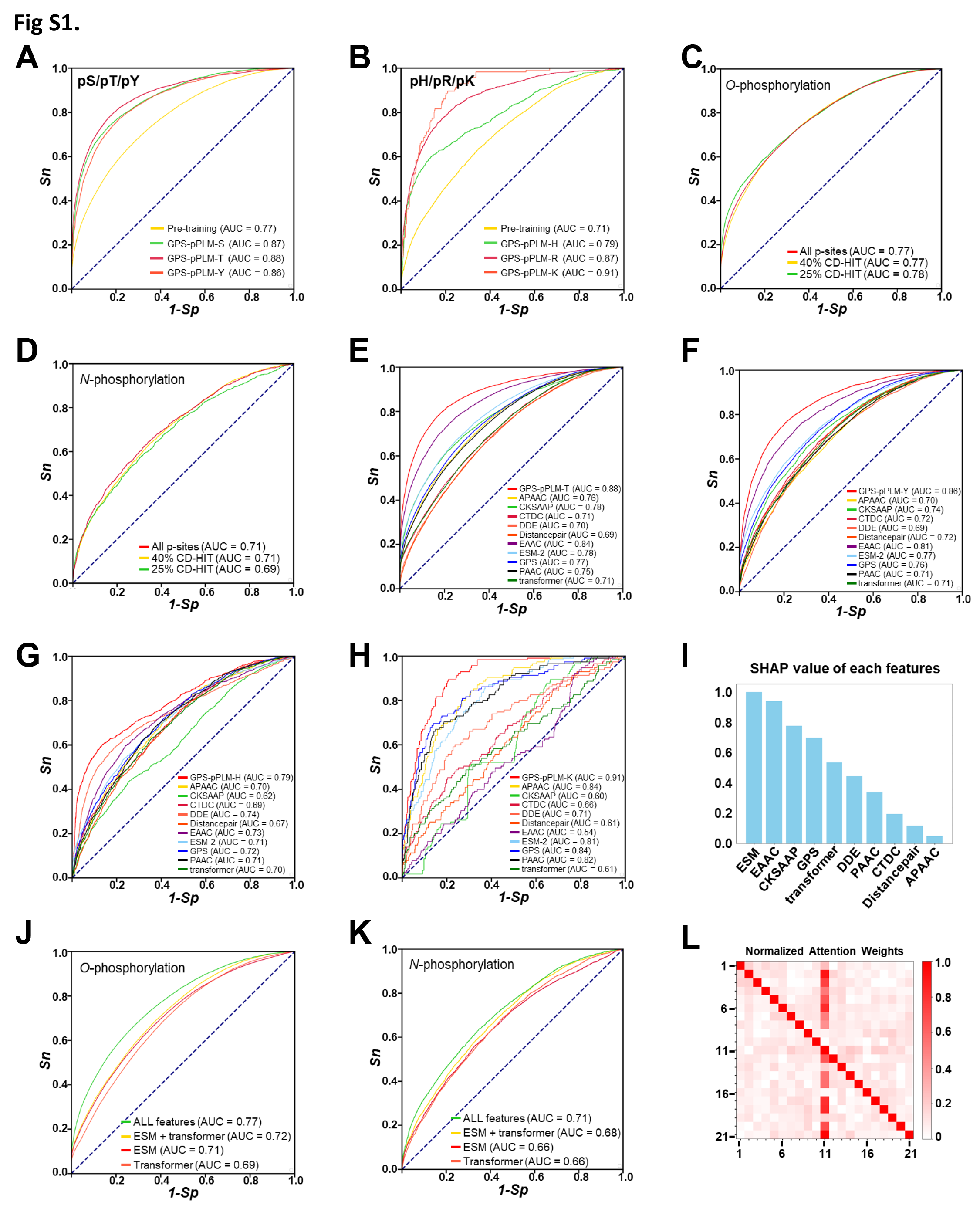

Supplement: Supplementary file 1 [file cells-13-01854-s001.zip › Supplementary Fig. S1.tiff]

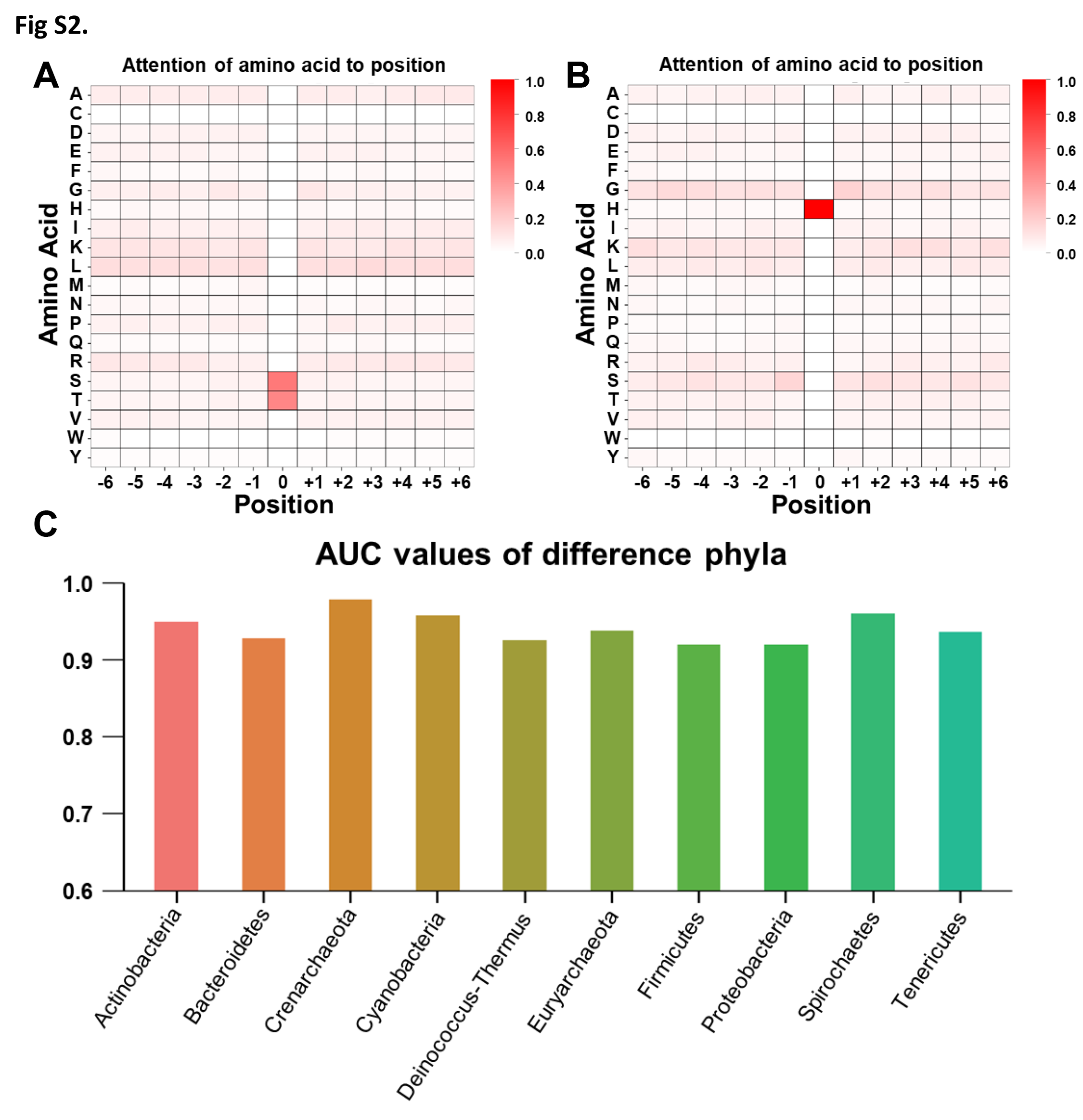

Supplement: Supplementary file 1 [file cells-13-01854-s001.zip › Supplementary Fig. S2.tiff]

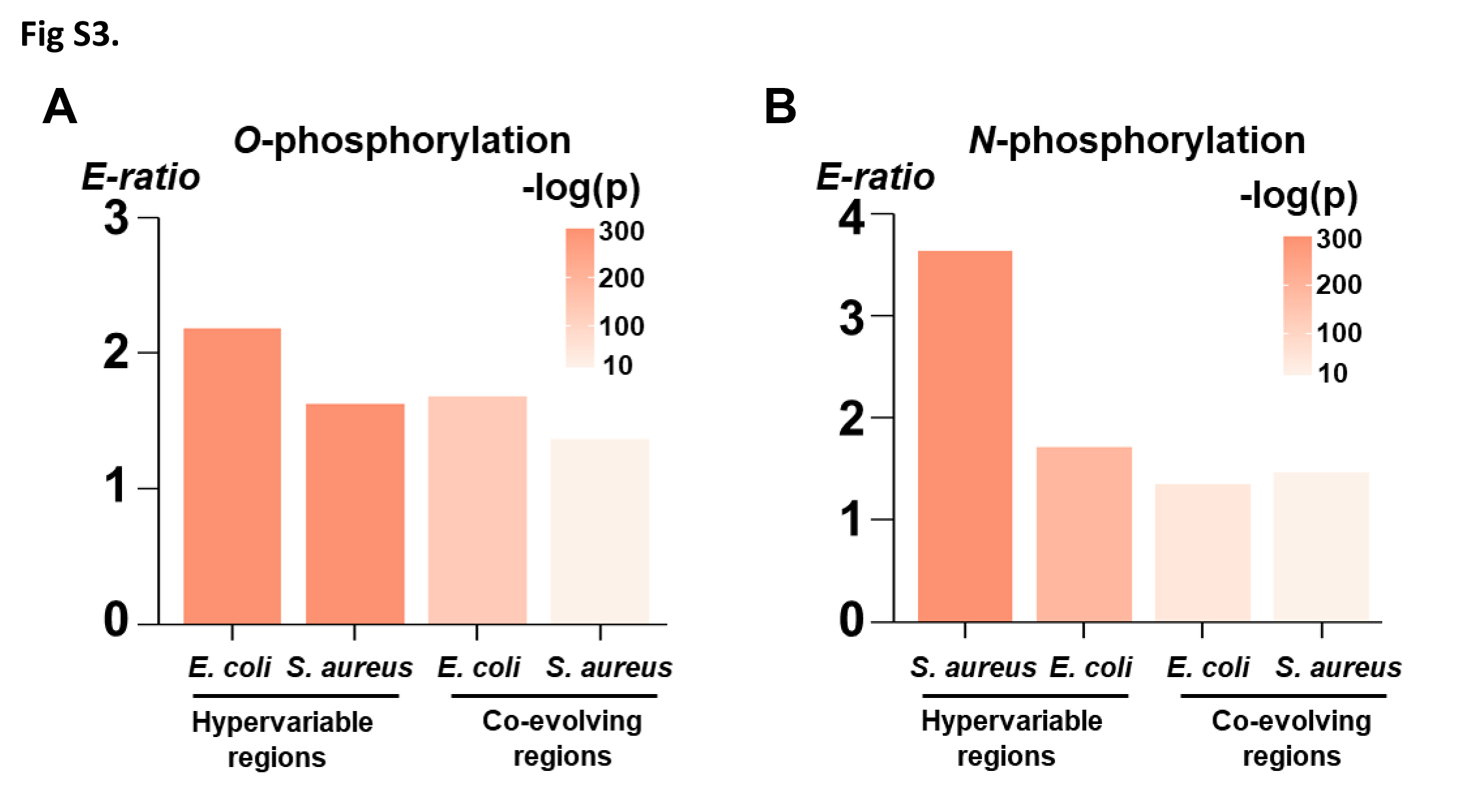

Supplement: Supplementary file 1 [file cells-13-01854-s001.zip › Supplementary Fig. S3.tiff]
